# Occurrence and Health Risk Assessment of Aflatoxins through Intake of Eastern Herbal Medicines Collected from Four Districts of Southern Punjab—Pakistan

**DOI:** 10.3390/ijerph18189531

**Published:** 2021-09-10

**Authors:** Aqib Javed, Iqra Naeem, Noreddine Benkerroum, Muhammad Riaz, Saeed Akhtar, Amir Ismail, Muhammad Sajid, Muhammad Tayyab Khan, Zubair Ismail

**Affiliations:** 1 Institute of Food Science and Nutrition, Bahauddin Zakariya University, Multan 60800, Pakistan; aqib.javed1102@gmail.com (A.J.); niqra5771@gmail.com (I.N.); riaz@bzu.edu.pk (M.R.); saeedbzu@yahoo.com (S.A.); tayyabkhan1122@gmail.com (M.T.K.); zubairismail001@gmail.com (Z.I.); 2 Canadian Food Inspection Agency, 93 Mount Edward Rd Charlottetown, Charlottetown, PE C1A 5T1, Canada; 3 Institute of Chemical Sciences, Bahauddin Zakariya University, Multan 60800, Pakistan; dr.msajid@bzu.edu.pk; 4 Nishter Medical Hospital, Multan 60800, Pakistan

**Keywords:** herbal medicine, aflatoxin, health risk, Punjab, exposure, cancer risk

## Abstract

Eastern herbal medicines (HMs) are plant-derived naturally occurring substances with minimum or no industrial processing that have long been used in traditional medicine. Aflatoxins are frequent contaminants of plants. Therefore, these mycotoxins are likely to contaminate HMs and pose a health risk to individuals using them on a regular basis as preventive or curative treatments of various diseases. The present study aimed to determine aflatoxin levels in the most popular Pakistani HM formulations and to assess the health risk associated with the intake of aflatoxins. A total of 400 samples of HM formulations collected from four districts of Punjab were analyzed for the quantification of aflatoxins, out of which 52.5% were found to be contaminated. The average daily dose (ADD) of AFB_1_ and AFs through the intake of HM formulations ranged between 0.00483 and 0.118 ng/kg bw/day and between 0.00579 and 1.714 ng/kg bw/day, respectively. The margin of exposure (MOE) and population cancer risk ranged from 99.49 to 29378.8 and from 0.00011 to 0.0325 liver cancer cases/10^5^ individuals/year (0.0075–2.455 liver cancer cases/10^5^ individuals/75 years), respectively. Despite the low exposure to aflatoxins from HM formulations in the four studied Punjab (Pakistan) districts, the frequent contamination of the analyzed samples suggests that official measures should be considered to manage the associated risk.

## 1. Introduction

Complementary and alternative medicines (CAMs), including traditional medicines (TMs), have long been practiced worldwide. The Eastern system of medicines (*Unani* medicine system) is an ancient TM system, which is widely used in South Asian countries, including India, Pakistan, Bangladesh, and Iran, and is also gaining acceptance in other parts of the world to prevent and cure various ailments. Around 65–80% of the population in developing countries principally rely on HMs for their day-to-day healthcare essentials [1,2]. The primary reasons behind the usage of TMs lie in that they are easily accessible at affordable prices, are patient-oriented, and are strongly related to patients’ beliefs. Additionally, being natural, this practice is believed by the general population to be safer and non-toxic compared with chemically synthesized alternatives [3,4].

Eastern herbal medicines (HMs), also known as *Tibb-i-Unani*, are plant-derived naturally occurring substances with minimum or no industrial processing that have been used to cure health issues within regional or local healing practices [5]. The plants and their parts, such as leaves, roots, bark, rhizomes, and flowers, contain several important chemical substances, including essential oils, alkaloids, terpenes, and vitamins that have specific therapeutic effects against several illnesses and for maintaining human health [6]. There has been exponential growth in the field of HMs in the last few decades. The global HM market was estimated to be around USD 83 billion in 2019 and is expected to reach USD 550 billion by 2030 [7]. Despite the ancestral use of HMs in traditional medicine, the evidence is a still insufficient regarding their safety aspects, particularly with regard to their contamination with mycotoxins, pesticide residues, toxic metals, polycyclic aromatic hydrocarbons, and alternate plant species [8].

Mycotoxins are toxic fungal metabolites primarily found in cereal grains, spices, nuts, traditional herbal products, and other produce and have been proven to cause several health conditions in both humans and animals. Mycotoxin contamination raises a serious food safety and public health concern that arguably has monetary impacts across the globe, particularly in low- and middle-income countries [9]. Aflatoxins are the most potent and widespread mycotoxins, which are mainly produced by fungi of the *Aspergillus* genus, particularly the species *A. flavus*, *A. paraciticus*, and *A. nomius* [10]. Among the more than 20 different types of aflatoxins identified so far, aflatoxin B_1_ (AFB_1_), aflatoxin B_2_ (AFB_2_), aflatoxin G_1_ (AFG_1_), and aflatoxin G_2_ (AFG_2_) are the most toxic and the most prevalent; they are thus referred-to as “major aflatoxins” [11]. Aflatoxin B_1_ and the sum of the four major aflatoxins (i.e., AFs) have been categorized as carcinogens of the group 1 category by the *International Agency for Research on Cancer (IARC)* [12] and have been associated with liver cancer in both humans and animals [13]. Ingestion of foods contaminated with aflatoxins results in its transformation into 8,9-epoxide metabolite in the liver or in triggering oxidative stress, with consequent severe health conditions [14]. In addition to being carcinogenic, aflatoxins have also been reported to be immunosuppressive, genotoxic, teratogenic, mutagenic, and growth retarding [14,15,16,17].

The Pakistani traditional system of medicines, which utilizes herbs and medicinal plants for the treatment of various diseases, is essentially based on the *Unani* system of medicine dating back to the Indus valley civilization spanning from 1300 to 2600 BCE [18,19]. Approximately 70–80% of the country’s population uses CAMs for healthcare purposes. More than 52,600 registered *Unani* medical practitioners are working both in the private and public sectors in rural and urban areas and in about 457 *Tibb* clinics and dispensaries, and 300–350 HMs/*Tibb-e-Unani*-producing industries are present in the country [20,21]. Moreover, Pakistan occupies a significant position in the international trade market of medicinal plants. The country ranks in the 10th position in the export of medicinal plants (8100 tons), and in the 9th position in imports (11,350 tons) [18,22].

While aflatoxins contamination of HMs has been reported in various countries from different parts of the world [23,24,25,26,27,28], limited data are available on the exposure and health risk assessment of aflatoxins in HMs. In Pakistan, the only study reported was restricted to the determination of aflatoxins in medicinal plants [29], and to the best of our knowledge, no study has been published on the determination of aflatoxins in Pakistani HM formulations or on the health risk to which they expose the Pakistani population. On the contrary, the high frequency of occurrence of aflatoxins in various food commodities and agricultural products in Pakistan at relatively high levels [30,31,32,33,34] compared with those reported worldwide [35,36,37,38] is well documented.

Mitigation of exposure to aflatoxins from different origins, including HMs, is an onerous, slow, and complex process involving official authorities, producers, consumers, scientists, national and international traders, and mass media, etc. This is even more tedious in the absence of, or in inadequately implemented, regulatory measures as key tools to gauge the extent of compliance of food commodities and agricultural products with the established safe levels and act accordingly. In Pakistan, only recently, the Punjab Food Authority (PFA) has set maximum tolerable limits (MTLs) for total AFs in all articles of food to 20 ng/g and for aflatoxin M_1_ (AFM_1_) in milk to 0.5 ng/g [39], which is highly permissive and yet loosely enforced [34].

The aim of the present study was to evaluate the occurrence of aflatoxins in the most commonly used HM formulations in four districts of Punjab, Pakistan. The potential exposure and health risk of aflatoxins resulting from consumption of aflatoxin-contaminated HM formulations was also evaluated.

## 2. Materials and Method

### 2.1. Sampling

A total of 400 samples of 20 HM formulation types produced in Pakistan (20 samples from each type) were purchased from local herbal medical stores located in four districts (Multan, Bahawalpur, Rahim Yar Khan, and Dera Ghazi Khan) of the province of Punjab (Pakistan). From each district, 100 samples were used in this study during the period of June to September 2020. The samples were packed in air-tight plastic bags and stored in a dark and dry place until analysis. Table 1 shows the types of HM formulations analyzed in this study, along with their composition, intake rate, and claimed therapeutic effects. The criterion used for selection was the extent of consumption in the region; only high consumption formulations were considered.

### 2.2. Sample Preparation and Aflatoxins Analysis

#### 2.2.1. Extraction and Immunoaffinity Clean-Up

The samples of HM formulations were extracted with 100 mL methanol/water (60/40 *v*/*v*) in an orbital shaker (Thermo Scientific, Waltham, MA, USA), MaxQ 4000) for 4–5 h at 200 rpm. The extracts were filtered using Whatman filter paper No. 42, and the resulting filtrates (4 mL) were diluted with 16 mL of 50 mM phosphate-buffered saline (PBS) having pH 7.4. The diluted extracts were passed through immunoaffinity columns (Eurofins, Siegen, Germany) according to the manufacturer’s recommendations.

#### 2.2.2. Post-Column Derivatization

The derivatization of aflatoxins was accomplished as stated by the AOAC official method 2005.08 [40]. Briefly, the dried extracts of samples and standards were dissolved in 200 μL of hexane and added with 50 μL trifluoro-acetic acid (TFA). The vials were then closed tightly and placed in the dark for 5–6 min before the addition of 1.95 mL mixture of double distilled water and acetonitrile (9:1) to each of them, followed by vortex mixing for 1–2 min. The lower aqueous layer containing aflatoxins was removed and filtered via syringe filter (0.45 μm) prior to chromatographic analysis.

#### 2.2.3. Chromatographic Analysis

A high-performance liquid chromatographic (HPLC) system, S 500 routine series using S1125 isocratic pump (Sykam, Eresing, Germany), coupled with a fluorescence detector (Sykam, RF-20A) was used. An isocratic mobile phase of water/methanol/acetonitrile (55/22.5/22.5 *v*/*v*/*v*) was used at a flow rate of 1.0 mL/min. A Welchrom (Welch Material, Inc., Austin, TX, USA) silica gel reverse phase C-18 column (4.6 × 250 mm) was used as a stationary phase. The run time for each standard and sample was 20 min, and the injection volume was 20 µL. The excitation and emission wavelengths of the fluorescence detector were 365 nm and 440 nm, respectively, and the column oven temperature was set at 37 °C. The retention times for AFB_1_, AFG_1_, AFB_2_, and AFG_2_ were 5.09, 4.28, 8.78, and 6.66 min, respectively.

#### 2.2.4. Method Validity

A mixed solution of aflatoxins in acetonitrile from Sigma-Aldrich (St. Louis, MO, USA) was used as a standard. The solution contained the four major aflatoxins: AFB_1_ (0.5 µg/mL), AFG_1_ (0.5 µg/mL), AFB_2_ (0.25 µg/mL), and AFG_2_ (0.25 µg/mL) *of* HPLC grade purity (≥98%) for each aflatoxin. Working standard solutions at four different concentrations (0.005, 0.010, 0.025, and 0.050 µg/mL) were prepared in acetonitrile and were used in recovery experiment and for preparation of calibration standards (solvent-matched). The recovery percentages were computed by spiking the aflatoxin-free samples of HMs at three different concentrations, 12, 24, and 48 µg/kg, with the ratios of AFB_1_, AFB_2_, AFG_1_, and AFG_2_ being 1.0:0.5:1.0:0.5, respectively. The standards of aflatoxins were quantified independently in nine replicates. The spiked samples were allowed to stand for 12 h to ensure the adsorption of aflatoxins within the samples before they were prepared for chromatographic analysis according to the procedure mentioned above. The recovery percentages were calculated by using Equation (1).

(1) $$\mathrm{Recovery}\;(\%)=\frac{\mathrm{measured}\;\mathrm{concentrations}}{\mathrm{spiked}\;\mathrm{concentrations}}\times 100$$

The recovery percentages for different types of aflatoxins ranged between 84.9% and 95.7%, while the variation co-efficient for aflatoxins ranged between 3.8% and 11.7%. The limit of detection (LOD) (3× standard deviation/slope) and the limit of quantification (LOQ) (3× LOD) were calculated according to the method described by Kortei et al. [41]. The LODs for AFB_1_, AFB_2_, AFG_1_, and AFG_2_ were 0.05 µg/kg, 0.03 µg/kg, 0.05 µg/kg, and 0.03 µg/kg, respectively. The LOQs for AFB_1_, AFB_2_, AFG_1_, and AFG_2_ were 0.15 µg/kg, 0.09 µg/kg, 0.15 µg/kg, and 0.09 µg/kg, respectively. All the experiments were performed in triplicates, and each sample was further analyzed three times to ensure the reliability of results.

### 2.3. Health Risk Assessment

#### 2.3.1. Exposure Assessment

The average daily dose (ADD) (expressed as ng/kg bw/day) of aflatoxins was computed based on the concentration of toxin detected and the intake rate of studied HM formulations. The HMs analyzed in the present study were a mixture of herbs, finely ground and suspended in water or milk at given ratios to be taken orally as recommended by the medical herbalist. The ADD, expressed in ng/kg bw/day, was calculated by using Equation (2) [42].

(2) $$\mathrm{ADD}=\frac{C\times \mathrm{IR}\times \mathrm{ED}\times \mathrm{EF}}{\mathrm{WAB}\times \mathrm{AT}}$$

where C is the concentration of aflatoxin (ng/kg). IR is the intake rate (kg of HM/day) calculated for children or adults according to the practitioner recommendations for the studied HM formulations (Table 1). EF is the exposure frequency; a figure of 90 days/year was used as recommended earlier [8,42,43,44,45]. ED is the exposure duration; 70 years was taken as the current average human lifespan. AT is the average time (ED × 365 days/year). W_AB_ is the average body weight; the respective values of 32.7 kg and 72 kg were used for Pakistani children and adults [46,47].

The left-censored data (data below LOD and LOQ) were processed by applying the substitution method of EFSA [48]. Two exposure scenarios were considered: a lower bound (LB) scenario, in which zero was assigned to samples showing aflatoxins concentration below LOD/LOQ, and an upper bound (UB) scenario, in which the value of LOD was assigned to the samples in which the aflatoxins concentration was below the detection limit, and the LOQ value was assigned to the samples where aflatoxins were present at levels below the LOQ [48].

#### 2.3.2. Health Risk Characterization

The risk characterization originating from the oral exposure to aflatoxins was computed using two approaches; the qualitative margin of exposure (MOE) approach established by EFSA for substances that are both genotoxic and carcinogenic [49] and the quantitative approach to liver cancer risk estimation proposed by the Joint FAO/WHO Expert Committee on Food Additives (JECFA) [50]. The MOE is the ratio between the point of departure (POD) for carcinogenesis, a toxicological reference point corresponding to a dose at which a low but measurable adverse response is first observed and the human exposure to the substance. The MOE value was calculated using Equation (3):

(3) $$\mathrm{MOE}=\frac{\mathrm{BMDL}10}{\mathrm{ADD}}$$

where BMDL_10_ is the benchmark dose lower confidence limit (BMDL_10_) for 10% increased cancer risk. The value of 170 ng/kg bw/day suggested by the EFSA on the basis of animal data with the application of uncertainty factors [51] was used in this study. ADD is the average daily dose used to estimate the exposure levels, as calculated in Equation (1).

Because substances that are both genotoxic and carcinogenic can pose health risks at any dose level, to explain the significance of our results, we followed the recommendation of JECFA and applied an MOE of 10,000.A calculated MOE value lower than 10,000 implies that the exposure to a carcinogenic and genotoxic substance is of concern to public health and should be given high-priority for risk management [52]. It should be mentioned, however, that the MOE value is not a measure of cancer risk per se, but it rather provides an estimation of the level of concern [49,53]. Therefore, the risk for liver cancer from AFB_1_ exposure via HM intake was calculated by a deterministic approach on the basis of AFB_1_ carcinogenic potency (P_cancer_) resulting from synergistic carcinogenic effects of hepatitis B virus (HBV) infection and AFB_1_, expressed by using Equations (4) and (5):

(4) $$P_{\mathrm{cancer}}\;=\left({\mathrm{PHBsAg}}^{+}\times \%\mathrm{Pop}.{\mathrm{HBsAg}}^{+}\right)+\left({\mathrm{PHBsAg}}^{-}\times \%\mathrm{Pop}.{\mathrm{HBsAg}}^{-}\right)$$

(5) $$\mathrm{Cancer}\;\mathrm{risk}=P_{\mathrm{cancer}}\;\times \mathrm{ADD}$$

where, P_cancer_ is the average carcinogenic potency of AFB_1_ expressed as the number of cancer cases per 100,000 individuals per ng of AFB_1_ per kg bw per day. PHBsAg^+^ and PHBsAg^−^ are P_cancer_ of aflatoxins for hepatitis B surface antigen-positive (HBsAg^+^) and hepatitis B surface antigen-negative (HBsAg^-^) individuals, respectively. The %Pop. HBsAg^+^ and %Pop. HBsAg^−^ are the prevalence of HBV and non-HBV carriers, respectively.

For HBsAg^+^ individuals, the P_cancer_ of AFs was 0.3 cancers/year/10^5^ individuals/ng AFB_1_/kg bw/day, while the P_cancer_ of AFs for HBsAg^−^ individuals was estimated to be 0.01 cancers/year/10^5^ individuals/ng AFB_1_/kg bw/day [54]. Considering the prevalence rate of 3.3% for HBsAg^+^ individuals reported by WHO for the Eastern Mediterranean Region [55], the P_cancer_ related to HMs intake in the four studied districts of Punjab (Pakistan) was estimated to be 0.019 cancers/year/10^5^ individuals/ng AFB_1_/kg bw/day. To calculate the cancer risk resulting from lifetime exposure (75 years), the resulting P_cancer_ value was then multiplied by 75 [54].

### 2.4. Statistical Analysis

The data were analyzed using Statistix 8.1 (Informer Tech. Inc., Los Angeles, CA, USA). All the measurements were performed in triplicates. A probability value (*p*-value) less than 0.05 was considered statistically significant. The data are expressed as mean ± standard deviation, computed using Microsoft Excel 2013 version. The analysis of variance (ANOVA) followed by Least Significance Difference (LSD) test was used for statistical comparison of the data. A Shapiro–Wilk test of normality was run to check the normality of data and after recording the data as normal, the analysis of variance (ANOVA) followed by a Least Significance Difference (LSD) test was used for statistical comparison of the data.

## 3. Results and Discussion

### 3.1. Aflatoxins Occurrence in Herbal Medicine Formulations

The results of aflatoxins occurrence in 400 samples of 20 HM formulations collected from four districts of the Punjab province (Pakistan) are summarized in Table 2, Table 3 and Table 4. Total AFs were detected in 52.5% of the analyzed samples, with AFB_1_ being the most frequently occurring (46.3%), followed by AFG_1_ (35.6%), AFB_2_ (34.5%), and AFG_2_ (27%) (Table 2). The results also demonstrate that there was a significant difference (*p* < 0.05) in the concentration of the five studied groups of aflatoxins (AFB1, AFB2, AFG1, AFG2, and AFs) among the HM formulations (Table 3), while no significant difference (*p* > 0.05) was found between aflatoxin levels in samples collected from the four districts of Punjab (Table 4). Table 3 summarizes the concentrations of the different types of aflatoxins in the analyzed samples of the 20 HM formulations. This table shows that AFs ranged from <LOD to 17.5 ng/g, with an average of 1.95 ng/g, while individual aflatoxins were detected in the range of <LOD–8.4 ng/g, <LOD–3.23 ng/g, <LOD–12.83 ng/g, and <LOD–8.93 ng/g with average values of 0.58 ng/g, 0.20 ng/g, 0.78 ng/g, and 0.39 ng/g for AFB_1_, AFB_2_, AFG_1_, and AFG_2_, respectively. As for the contamination levels per type of HM, the highest average level of AFs (12.40 ng/g) was recorded in the Safoof-e-Lal, followed by Safoof-e-Mughaliz (4.21 ng/g) and Allergex (3.26 ng/g), while none of the analyzed aflatoxin types was detected in Akhseer-e-Pachish and Johar Hazim samples by the technique used (Table 3). It is worth mentioning that AFB_1_ and AFs concentrations exceeded the EU MTLs of 2 ng/g and 4 ng/g in 5.75% (n = 23) and 10.25% (n = 41) of HMs samples, respectively (Table 5) [56,57]. However, the average concentrations of AFB_1_ and AFs in the studied samples of HM formulations altogether were below the abovementioned MTLs; yet, the average concentration of AFs in each of two formulations, Safoof-e-Lal and Safoof-e-Mughaliz, exceeded the MTL of the EU and the MTL of the European Commission of 10 ng/g for spices ([58] Section 2.1.9) in one formulation, Safoof-e-Lal (Table 3), but they remained below the Pakistani MTL of 20 ng/g for all articles of food [34]. Moreover, the prevalence of AFB_1_ and AFs in the samples of HM formulations in the four districts varied between 22% and 60%, depending on the aflatoxin type, with average concentrations of 0.5 to 0.63 ng/g for AFB_1_ and 1.8 to 2.2 ng/g for AFs (Table 4).

Occurrence of aflatoxins in Eastern Medicines/HMs have been reported in various parts of the world. In Southeastern Nigeria, 84.21% of 57 studied Eastern Medicines were found to be contaminated with AFs at levels varying from below the LOD to 20 ng/g, with an average concentration of 7.35 ng/g [23]. A Korean study also demonstrated that out of 700 analyzed samples of HMs, 2.43% (n = 17) were positive for AFs, with concentrations ranging between 4.51 and 108.42 ng/g and that 35.29% of the positive samples exceeded the regulatory limit of 10 ng/g set by Korean Food and Drug Administration [59]. Zhao et al. [60] analyzed 22 samples of Chinese HMs for the presence of aflatoxins and found that 63.6% (n = 14) of them were contaminated; AFs and AFB_1_ concentrations varied from 0.2 to 7.5 ng/g and 0.2 to 4.8 ng/g, respectively. Another study of 20 samples of medicinal plants used for HM preparations in China reported the occurrence of AFs at a rate of 40%, with 10% (n = 2) exceeding the regulatory Chinese limit [61]. In India, Afs content in crude medicinal plants used for herbal medicine formulations ranged between <LOD and 24 ng/g. Ali et al. [62] reported that the average concentrations of AFB_1_, AFB_2_, AFG_1_, and AFG_2_ in 23 samples of traditional herbal medicine preparations from Malaysia and Indonesia were 0.26 ng/g, 0.07 ng/g, 0.10 ng/g, and 0.03 ng/g, with a prevalence of 70%, 61%, 30%, and 4%, respectively. An analysis of 16 samples of HMs from South Africa revealed that the concentration of AFs in all of the analyzed samples was below the detectable level of 0.5 ng/g [24]. Nonetheless, such results may not be conclusive due to the low number of samples analyzed. In comparison with previous studies, the levels of aflatoxins found in the Pakistani HM formulations in this study fell in the same range as those reported in Thai HMs, where 18% of the samples were contaminated with levels varying between 1.7 and 14.3 ng/g [25]. The level of contamination of HM preparations made with organic medicinal plants may be higher than that of those made from conventional medicinal plants due to the restricted use of synthetic fungicides in organic agriculture. This was demonstrated in Turkish samples of different herbs commonly used in traditional medicine, where 86% of the analyzed samples were contaminated with AFB_1_, at mean levels varying between 5.7 and 44.5 ng/g, depending on the herb, and the percentage of the positive samples varied from 60% to 100%, with 65.6% of the positive samples (21 out of 32 samples) exceeding the EU MTL [28]. Conversely, our result are lower than those reported in HMs from Southeastern Nigeria [23], South Korea [59], and India [61] and are higher than those from China [60], South Africa [24], and Indonesia and Malaysia [62]. Many factors may account for discrepancies regarding aflatoxin contamination of HMs from different countries; these include the sampling season and procedure, the constituents of HM formulations, the varieties and chemotypes of raw medicinal plants, the climate and soil of the region where these plants grew, and the post-harvest conditions of preparation, packaging, and storage. In fact, within the same region the extent of contamination may vary greatly from one formulation to another. For example, contrary to the rest of the HM formulations studied herein, none of the five types of aflatoxins were detected in Akhseer Pachish and Johar Hazim formulations. On the contrary, samples of Safoof-e-Thandak, Majoon Azaraqi, Safoof-e-Lal formulations were contaminated with the highest levels of the five aflatoxin types. In Safoof-e-Lal and Safoof-e-Mughaliz formulations, the total AFs content exceeded the most stringent MTL of 4 ng/g set by the *European Pharmacopoeia* [56] (Table 3). Apart from environmental parameters and agricultural practices, such variations between HM formulations may be ascribed to the conditions of preparation, packaging, and storage by herbalists, which may account for the high contamination of some medicinal plants, despite their well-documented resistance to mold growth and/or toxigenesis [63,64,65,66], owing to their ability to produce antifungal bioactive substances [67,68,69].

### 3.2. Health Risk Assessment

#### 3.2.1. Exposure Assessment

The exposure of the south Punjab (Pakistan) population (children and adults) to AFB_1_ and to total AFs from the intake of HM formulations was assessed by the ADD determinations; the results are summarized in Table 6. Irrespective of the age and for both lower bound (LB) and upper bound (UP) scenarios, the ADD of AFB_1_ and total AFs through the consumption of HM formulations ranged between 0.01 and 0.12 ng/kg bw/day and between 0.01 and 1.71 ng/kg bw/day, respectively. For children, the mean LB exposure to AFB_1_ and total AFs ranged from 0.01 to 0.11 ng/kg bw/day and from 0.01 to 1.40 ng/kg bw/day, and the mean UB exposure varied from 0.02 to 0.12 ng/kg bw/day and from 0.01 to 1.41 ng/kg bw/day, respectively. For adults, the mean LB exposure to AFB_1_ and total AFs ranged from 0.003 to 0.11 ng/kg bw/day and from 0.01 to 1.70 ng/kg bw/day, and the mean UB exposure from 0.01 to 0.11 ng/kg bw/day and from 0.01 to 1.71 ng/kg bw/day, respectively. The average ADD of total AFs through consumption of HM formulations was higher for children (0.21 and 0.22 ng/kg bw/day for the LB and the UB, respectively) than for adults (0.17 and 0.18 ng/kg bw/day for LB and UB, respectively). This can be explained by the lower body weight of children compared with adults, which outweighs the effect of the intake rate. As regards the type of medicine, Safoof-e-lal contributed the highest level of exposure to total AFs (1.55 ng/kg bw/day), followed by Alhazim (0.16 ng/kg bw/day) and Senna Maki (0.13 ng/kg bw/day) (data not shown). Overall, none of the samples analyzed exceeded the respective MTLs of 2 ng/g and 4 ng/g for AFB_1_ and AFs set by *European Pharmacopoeia* [56,70] or those set by the *United States Pharmacopeia* (USP) of 5 ng/g for AFB_1_ and 20 ng/g for total AFs. Such results suggest that the exposure of the Southern Punjab Pakistani population to aflatoxins from HM formulations is too low to raise a serious public health concern. The risk may be even lower if the HM formulations were administered as infusions, where the plant material is separated from the beverage to be taken after the infusion process. This treatment of medicinal plants was reported to reduce aflatoxin content by 70% to 100% [71]. Nonetheless, our results indicate that aflatoxins are rather common contaminants of HM formulations in the studied region of Punjab, with 46.3% and 52.5% for AFB_1_ and total AFs, respectively (Table 2). The growing use of medicinal plant preparations in popular medicine in different countries around the world, and particularly in developing countries where they are usually informally marketed beyond any official control, is an additional risk factor [72]. Moreover, due to the inconsistent harvest, preparation, distribution, and storage conditions of HMs, significantly higher levels of aflatoxins in HMs from remote areas can be reasonably anticipated. Therefore, the potential risk that the consumption of such medicines pose to public health cannot be ruled out, especially for consumers in the 95th percentile if these products continue to be marketed without official control. Meanwhile, it is recommended that the health risk associated with aflatoxins in HMs in Pakistan be systematically assessed to serve as a scientific basis for the development of adequate regulatory standards. Although, no tolerable daily intake (TDI) can be used to define safe levels as a reference for putative regulations due to the lack of a threshold response of aflatoxins as carcinogenic and genotoxic toxicants, the “as low as reasonably achievable” (ALARA) approach can be adopted to ensure safe use of HM formulations. More comprehensive surveys of the prevalence and extent of contamination of HMs with aflatoxins are needed to provide sufficient data for a meaningful risk characterization, and hence to provide a sound basis for regulatory provisions [52]. Few studies, to our knowledge, have conducted a formal risk assessment of aflatoxins in medicinal plants, their formulations, or their extracts/infusions in other countries. Pallarés, Berrada, Fernández-Franzón and Ferrer [71] surveyed 224 samples of 56 different species of medicinal plants commercialized in Spain for the occurrence and the level of contamination with different mycotoxins, including the four major aflatoxin types (AFB_1_, AFB_2_, AFG_1_, AFG_2_). The study showed that aflatoxins were found in the raw materials at mean concentrations ranging between 64.76 and 838.58 ng/g for the major aflatoxins; however, their prevalence and concentrations were drastically reduced in the infusions with the notable elimination of AFB_1_. The authors concluded that the health risk associated with the consumption of medicinal plant infusions was low, and yet, it should be managed with the ALARA approach. However, no inference in the study was made to the risk associated with the ingestion of the raw medicinal plants. Similar results were reported on Moroccan aromatic and medicinal plants, whose infusions were shown not to pose an appreciable health risk owing to a low exposure, in spite of the fact that the concentrations of total AFs in some raw plant material exceed the regulatory standards of 4 ng/g or 10 ng/g [73]. It is worth mentioning that AFB_1_, the most toxic aflatoxin, whose carcinogenicity for humans is well established, was not detected in any of the samples analyzed in the latter study. Exposure of Nigerian infants and young children (IYC) to abnormally high health risks from the consumption of aflatoxin-contaminated complementary foods with too low MOE values (0–70 for AFB_1_ and 0–7 for AFs) [37] or a too high exposure (641 ng/kg bw per day) [36] was reported. However, in the latter studies, the main ingredients, e.g., maize, rice, oat, wheat, millet, and peanut of the surveyed foods are notoriously known for their vulnerability to contamination with various mycotoxins. For example, AFB_1_ concentration reached a value as high as 51,192 ng/g in *Tom bran*, a whole meal from mixed grains, including maize and peanut [36]. This may account for the difference in our results on HM preparations, generally consisting of a mixture of medicinal plants with varying degrees of antifungal activities [69].

#### 3.2.2. Health Risk Characterization

The findings of the characterization of the risk for hepatocellular carcinoma (HCC) development upon exposure to AFs based on the MOE approach using ADD and BMDL_10_ as well as by P_cancer_ and ADD are presented in the Table 7. The average overall MOE values obtained for total AFs exposure through consumption of HM formulations ranged from 99.5 to 29,378.8. For children, the mean LB MOE for total AFs ranged from 121 to 26,680, and the mean UB MOE values ranged from 120 to 17,409. For adults, the mean LB MOE values ranged from 100.1 to 29,372.8, and the mean UB MOE values ranged from 99.5 to 19,166.0. The MOE values calculated for each medicine type were far below the safe margin of 10,000 suggested by EFSA [52], with the exception of Khameera Marvareed, for which the MOE values for the LB scenario (28,026.55) and the UP scenario (18,287.55) were higher than the safe margin of 10,000, indicating that the exposure to this particular HM formulation poses a low health risk. Conversely, MOE values calculated for of the rest of the HM formulations indicate that they expose Punjabi users to a high risk, and hence they require official management measures.

The mean overall estimated number of liver cancer cases based on the consumption of HM formulations ranged between 11 × 10^−5^ and 3.25 × 10^−2^ liver cancer cases per 10^5^ individuals per year and 75 × 10^−4^ and 2.5 × 10^−1^ liver cancer cases per 10^5^ individuals per 75 years. For the child population, the mean estimated number of liver cancer cases for LB scenario ranged from 11 × 10^−5^ to 264 × 10^−4^ cases/10^5^ individuals/year (71 × 10^−4^ to 1.951 cases/10^5^ individuals/75 years) and for UB scenario ranged from 18 × 10^−5^ to 268 × 10^−4^ cases/10^5^ individuals/year (135 × 10^−4^ to 2.010 cases/10^5^ individuals/75 years). For the adult population, the mean estimated number of liver cancer cases for the LB scenario ranged from 1 × 10^−4^ to 321 × 10^−3^ cases/10^5^ individuals/year (75 × 10^−4^ to 2.5 cases/10^5^ individuals/75 years) and for UB scenario ranged from 2 × 10^−4^ to 325 × 10^−4^ cases/10^5^ individuals/year (127 × 10^−4^ to 2.5 cases/10^5^ individuals/75 years). As regards the type of medicine, the intake of Safoof-e-Lal contributed a higher risk of HCC cases/10^5^ individuals/year (0.026(LB)–0.3455(UB)), followed by Alhazim (0.0028(LB)–0.0033(UB)) and Hazmina Plus (0.0019(LB)–0.0029(UB)). The estimated number of liver cancer cases associated with the lifetime exposure to AFs through consumption of these HM formulations was higher than the proposed limit of 0.1 cases/10^5^ individuals/75 years [54,74]. Such results indicate that the lifetime exposure to AFs through consumption of HM formulations is a matter of concern for the health of the child and adult population of Pakistan.

## 4. Conclusions

Despite the growing use of traditional medicine with HM formulations in Pakistan, no studies have been conducted on the potential risk they may pose to public health regarding their contamination with aflatoxins. This was the first study to investigate the occurrence of aflatoxins in 20 of the most commonly used HM formulations in four districts of Punjab (Pakistan) in order to perform a preliminary risk assessment of which population would be exposed in the region. Our results show that more than 46% and 50% of the analyzed samples were positive for total aflatoxins (AFs) and the most potent aflatoxin, AFB_1_, respectively. Although, generally low, the average concentrations of AFB_1_ and total AFs were higher than the EU regulations in one and two HM formulations, respectively. Additionally, at the individual level, 5.7% and 10.25% of samples exceeded the latter MTL of AFB_1_ and total AFs, respectively. The exposure data suggest that children are more at risk than adults, mainly because of their lower bodyweight. Although the overall health risk of aflatoxins and the calculated annual rate of extra-liver cancer cases caused by consumption of HM formulations were low, the risk may still be of concern, particularly with continuous exposure of the heavy consumers (95th percentile), and cannot be ignored. Further investigations are thus required, including the implementation of an adequate surveillance system and long-term monitoring of aflatoxin contamination in as many HM formulations and medicinal plants as possible. Thorough surveys of the extent and frequency of consumption of these products are also necessary to provide a more realistic risk assessment of outcomes, taking into account different consumption patterns, groups, and the percentiles of consumers. Studies to evaluate the co-occurrence of potentially harmful mycotoxins in the herbal medicine formulations and their synergistic or antagonistic effects can help refine any risk assessment conducted on aflatoxins as standalone hazards. Meanwhile, regulatory measures based on previously established MTLs in different countries can be issued to ensure the safety and quality of herbal medicines.

## Figures and Tables

**Table 1:** General information of selected herbal medicine formulations (HMs) consumed in Pakistan.

HM No	Medicine Name	Composition	Target Population	Recommended Dosage		Therapeutic Indications
				Children	Adults	
1	Hazmina Plus	*Carcum copticum*, *Piper nigrum*, *Mangifera indica*, *Zingiber officinale*, ammonium chloride, black salt	children and adults	5 g twice in a day	10 g thrice in a day	Helps to control dyspepsia, eases in digestion, flatulence, heaviness in abdomen, and irritable bowel syndrome.
2	Safoof-e-lal	*Myrobolan green*, liquorice root, *Pistacia integerrima*, *Orchis latifolia Linn*, *Viola odorata*	children and adults	5 g thrice in a day	10 g four times in a day	Expectorant for respiratory disorder, shortness of breath, dryness of bronchial airways, irritation, cough, cold, and flu.
3	Safoof Musaffi-e-Khoon	*Terminalia chebula*, *Calotropis procera*, *Cassia fistula*	children and adults	5 g once in a day	10 g once in a day	Effective blood purifier, cures pimples, boils, and other skin eruptions.
4	Safoof Mumanek Khas	*Orchis latifolia*, *Dactylorhiza hatagirea*, *Sida cordifolia*, *Chlorophytum borivilianum*	adults	NR	10 g once in a day	Strengthens the reproductive system and effective in muscle cramp.
5	Safoof Lecodine	*Punica granatum*, *Amaranthus viridis*, *Citrullus colocynthis*, *Fumaria officinalis*	adult females	NR	5 g thrice in a day	Effective in leucorrhea and amenorrhea.
6	Akhseer Pachish	*Plantago ovata* husk, *Punica granatum*, *Chrysopogon zizanioides*, *Polygonum aviculare*	children and adults	5 g thrice in a day	10 g thrice in a day	Effective in acute and chronic diarrhea, griping, intestinal irritation, dysentery, and mucus and bloody stools.
7	Johar Hazim	*Illicium verum*, *Hyoscyamus niger*, *Piper longum*	children and adults	5 g thrice in a day	5 g thrice in a day	Effective in stomach acidity, irritable bowel syndrome, improves digestion.
8	Khameera Marvareed	*Mytilus margaritiferus*, *Vateria Indica, Bambusa arundinacea, Santalum Album*, Serpentine, Sugar	children and adults	5 g once in a day	10 g once in a day	Potent cardiac tonic, relieves perplexity and palpitation, useful in measles, normalizes high blood pressure.
9	Safoof-e-Thandak	liquorice root, *Cucumis melo* seeds, *Trachyspermum ammi*, Tragacanth gum, *Portulaca oleracea*, *Althaea officinalis*, *Prunus dulcis*	children and adults	5 g twice in a day	10 g twice in a day	Effective in bladder inflammation, burning and dysuria, controls the intrinsic heat of the body, normalizes the effects of heat, sunstroke, and thirst.
10	Safoof-e-Supari pak	*Areca catechu*, *Rubia cordifolia*, *Tribulus terrestris*, *Butea monosperma*, *Cinnamomum zeylanicum*, *Amomum subulatum*, *Zingiber officinalis*, *Asparagus adscendens*, *Syzyglum aromaticum*, *Myristica fragrans*, *Bombex malabaricum*, *Pistacla vera*, *Acacia nilotica*, *Bouhinia variegata*, *Censcora decussate*	adult females	NR	10 g once in a day	A specific remedy for leucorrhea, effective in general weakness, paleness, blood deficiency, muscular and nervine, weakness associated with leucorrhea, tones up uterus, and stops excessive fluid discharge.
11	Gond Katira	*Valeriana officinale*, *Pistacia lentiscus*, Red ochre, *Orchis latifolia*, *Pastinaca secaucus*, *Wrightia tinctoria*, *Bergenia ligulata*, *Punica granatum*, *Butea monosperma*, *Cochlospermum religiosum*, *Vachellia nilotica*, *Salvia haematodes*	children and adults	5 g twice in a day	10 g thrice in a day	Effective in hepatitis, urinary problems, maintains nocturnal ejaculation, gonorrhea.
12	Safoof-e-Mughaliz	Babul pods, liquorice, Austral sage, *Vachellia nilotica*	adult males	NR	10 g once in a day	Increase and thicken the seminal fluid, cures nocturnal emission and spermatorrhea. Additionally produces vigor, vitality, and virility by strengthening the nerves.
13	Safoof-e-Tabkhir	*Trachyspermum ammi*, *Foeniculum vulgare*, *Plantago ovata* husk, *Cuminum cyminum*, liquorice root, *Mentha arvensis*, sodium chloride, *Elettaria cardamomum*, sal ammoniac, *Coriandrum sativum*	children and adults	5 g once in a day	10 g once in a day	Indigestion, acidity, and associated problems such as flatulence, heart burn, vertigo, vomiting, and stomachache.
14	Allergex	*Coriandrum sativum*, *Mentha arvensis*, *Foeniculum vulgare*, brown sugar	children and adults	5 g once in a day	10 g once in a day	Effective in allergies due to intrinsic heat, medicines, and food conditions such as allergic dermatitis, urticaria, itching, genital pruritis, and eczema.
15	Safoof-e-Jarian	Liquorice root, *Vachellia nilotica*, hydrated magnesium silicate, *Argyreia nervosa*	adult males	NR	10 g once in a day	Effectively removes the causes of spermatorrhea and premature ejaculation. Thickens the seminal fluid and eliminates its unnatural and unwilling discharge. Additionally useful in regaining vitality and vigor.
16	Senna Maki	*Senna alexandrina*, *Nigella sativa*, *Piper nigrum*, Rosa, *Zingiber officinale*, Roscoe	children and adults	5 g twice in a day	10 g twice in a day	Effective in constipation, fever, asthma, dyspepsia, obesity, bone problems.
17	Kalvanji Hazim	*Nigella sativa*, *Trachyspermum ammi*, *Cuminum cyminum*, *Sodium bicarbonate*, *Senna alexandrin*	children and adults	5 g twice in a day	10 g twice in a day	Effective in nausea, constipation, dyspepsia, heart burn.
18	Majoon Azaraqi	*Strychnos nux-vomica*, *Onosma bracteatum*, *Hyssopus officinalis*, Astragalus gummifer gum, *Pinus Gerardiana*, *Lodoicea maldivica* fruit pulp, *Lavandula stoechas*, *Emblica officinalis*, *Elettaria cardamomum* seed, *Terminalia chebula*, *Pastinaca secacul*, *Curcuma zedoaria* root, *Aquilaria agallocha* fungus, *Caryophyllus aromatica* bud, honey	adults	NR	10 g once in a day	Effective for nervine weakness and neuromuscular pain; useful in paralysis, tremors, and rheumatic pain; regulates the digestive system; nervine stimulant, cardiac stimulant, analgesic, anti-inflammatory; urinary bladder tonic, anti-gout, antiepileptic, anticonvulsant, aphrodisiac.
19	Alhazim	*Piper longum*, *Piper nigrum*, *Zingigiber officinale*, *Carcum capticum*, *Cuminum cyminum*, *Mangifera indica* powder, *Mentha arvensis*, Black salt.	children and adults	5 g thrice in a day	10 g thrice in a day	Effective in abdominal pain, constipation, vomiting, nausea, stomach ulcers.
20	Habis	Red ochre, Shellac, *Shorea robusta*, aluminum potassium sulfate, *Butea monosperma*, *Dracaena cinnabari*, *Saussurea costus*, hydrated magnesium silicate	adult females	NR	5 g thrice in day	Effective in trauma, wound, menorrhea, hemorrhoid, and every type of bleeding, hemoptysis, epistaxis, and hematuria.

NR = not recommended (herbal medicine is not for consumption by that particular group of age).

**Table 2:** Occurrence of different types of aflatoxins in 20 analyzed samples of each of 20 different HM formulations collected from four Punjabi provinces of Pakistan.

HM formulation	P (%P)				
	AFB_1_	AFB_2_	AFG_1_	AFG_2_	AFs
Hazmina Plus	12 (60)	12 (60)	04 (20)	02 (10)	12 (60)
Safoof-e-Lal	13 (65)	07 (35)	18 (90)	18 (90)	18 (90)
Safoof Musaffi-e-Khoon	9 (45)	6 (30)	8 (40)	6 (30)	15 (75)
Safoof Mumanek Khas	15 (75)	14 (70)	15 (75)	4 (20)	15 (75)
Safoof Lecodine	5 (25)	3 (15)	5 (25)	0 (0)	5 (25)
Akhseer Pachish	0 (0)	0 (0)	0 (0)	0 (0)	0 (0)
Johar Hazim	0 (0)	0 (0)	0 (0)	0 (0)	0 (0)
Khameera Marvareed	2 (10)	0 (0)	1 (5)	0 (0)	2 (10)
Safoof-e-Thandak	15 (75)	15 (75)	14 (70)	14 (70)	15 (75)
Safoof-e-Supari Pak	15 (75)	15 (75)	8 (40)	7 (35)	15 (75)
Gond Katira	14 (70)	8 (40)	8 (40)	7 (35)	14 (70)
Safoof-e-Mughaliz	7 (35)	6 (30)	13 (65)	13 (65)	14 (70)
Safoof-e-Tabkhir	12 (60)	9 (45)	11 (55)	10 (50)	12 (60)
Allergex	14 (70)	10 (50)	8 (40)	14 (70)	14 (70)
Safoof-e-Jarian	16 (80)	16 (80)	13 (65)	3 (15)	16 (80)
Senna Maki	06 (30)	3 (15)	3 (15)	3 (15)	6 (30)
Kalvanji Hazim	11 (55)	2 (10)	5 (25)	0 (0)	13 (65)
Majoon Azaraqi	4 (20)	2 (10)	4 (20)	2 (10)	7 (35)
Alhazim	9 (45)	9 (45)	2 (10)	2 (10)	10 (50)
Habis	6 (30)	1 (5)	3 (15)	3 (15)	7 (35)
Total	185 (46.3)	138 (34.5)	142.4 (35.6)	108 (27)	210 (52.5)

P = Positive samples (>LOD); %P = percentage of positive samples (i.e., prevalence). AFB1 = Aflatoxin B1; AFB_2_ = Aflatoxin B_2_; AFG_1_ = Aflatoxin G_1_; AFG_2_ = Aflatoxin G2; Afs = sum of AFB_1_, AFB_2_, AFG_1_, and AFG_2_.

**Table 3:** Mean concentrations (±standard deviation) of aflatoxins (ng/g) in 20 selected herbal medicine formulations from Pakistan.

HM formulation	AFB_1_	AFB_2_	AFG_1_	AFG_2_	AFs
Hazmina Plus	0.88 ± 0.70 ^ab^	0.36 ± 0.43 ^bc^	0.13 ± 0.25 ^c^	0.02 ± 0.05 ^c^	1.39 ± 1.29 ^cde^
Safoof-e-Lal	0.77 ± 0.751 ^ab^	0.20 ± 0.32 ^bcde^	9.12 ± 3.07 ^a^	2.32 ± 1.30 ^a^	12.40 ± 3.83 ^a^
Safoof Musaffi-e-Khoon	0.50 ± 0.65 ^abcde^	0.22 ± 0.40 ^bcdef^	0.24 ± 0.33 ^c^	0.39 ± 0.71 ^bc^	1.34 ± 1.29 ^cde^
Safoof Mumanek Khas	0.79 ± 0.82 ^ab^	0.29 ± 0.17 ^bcd^	0.33 ± 0.24 ^c^	0.08 ± 0.13 ^c^	1.49 ± 1.03 ^cd^
Safoof Lecodine	0.32 ± 0.36 ^bcde^	0.07 ± 0.08 ^def^	0.28 ± 0.29 ^c^	<LOD	0.67 ± 0.70 ^cde^
Akhseer Pachish	<LOD	<LOD	<LOD	<LOD	<LOD
Johar Hazim	<LOD	<LOD	<LOD	<LOD	<LOD
Khameera Marvareed	0.14 ± 0.41 ^cde^	<LOD	0.03 ± 0.13 ^c^	<LOD	0.17 ± 0.52 ^de^
Safoof-e-Thandak	0.86 ± 1.08 ^ab^	0.32 ± 0.37 ^bcd^	0.47 ± 0.61 ^c^	0.33 ± 0.41 ^c^	1.98 ± 2.35 ^c^
Safoof-e-Supari Pak	0.91 ± 0.77 ^a^	0.37 ± 0.29 ^a^	0.20 ± 0.26 ^c^	0.17 ± 0.26 ^c^	1.66 ± 1.34 ^c^
Gond Katira	0.79 ± 0.68 ^ab^	0.15 ± 0.21 ^cdef^	0.14 ± 0.22 ^c^	0.23 ± 0.29 ^c^	1.40 ± 0.93 ^cde^
Safoof-e-Mughaliz	0.08 ± 0.11 ^de^	0.07 ± 0.10 ^ef^	3.23 ± 2.69 ^b^	0.82 ± 0.68 ^b^	4.21 ± 3.15 ^b^
Safoof-e-Tabkhir	0.83 ± 0.85 ^ab^	0.21 ± 0.26 ^bcde^	0.30 ± 0.37 ^c^	0.14 ± 0.16 ^d^	1.48 ± 1.40 ^def^
Allergex	0.67 ± 0.70 ^abc^	0.29 ± 0.38 ^bcd^	0.28 ± 0.45 ^c^	2.02 ± 1.88 ^a^	3.26 ± 2.87 ^b^
Safoof-e-Jarian	0.74 ± 0.71 ^ab^	0.59 ± 0.40 ^a^	0.35 ± 0.49 ^c^	0.09 ± 0.20 ^c^	1.76 ± 1.37 ^c^
Senna Maki	0.53 ± 0.62 ^abcde^	0.21 ± 0.20 ^bcdef^	0.18 ± 0.30 ^c^	0.85 ± 1.17 ^b^	1.77 ± 1.88 ^c^
Kalvanji Hazim	0.78 ± 0.99 ^ab^	0.05 ± 0.11 ^ef^	0.08 ± 0.15 ^c^	<LOD	0.92 ± 1.00 ^cde^
Majoon Azaraqi	0.60 ± 1.33 ^abcd^	0.17 ± 0.37 ^bcdef^	0.25 ± 0.57 ^c^	0.19 ± 0.43 ^c^	1.21 ± 2.55 ^cde^
Alhazim	0.99 ± 1.26 ^a^	0.37 ± 0.46 ^ab^	0.05 ± 0.11 ^c^	0.04 ± 0.09 ^c^	1.45 ± 1.79 ^cd^
Habis	0.44 ± 0.76 ^abcde^	0.03 ± 0.06 ^ef^	0.06 ± 0.12 ^c^	0.08 ± 0.16 ^c^	0.60 ± 0.93 ^cde^
Total *	0.58 ± 0.68	0.20 ± 0.23	0.78 ± 0.53	0.39 ± 0.40	1.95 ± 1.51

Results are expressed as mean of 20 determinations ± standard deviation; statistically significant difference was observed among means having different letters within the columns (*p* < 0.05); AFs is the sum of AFB_1_, AFB_2_, AFG_1_, and AFG_2_; LOD = limits of detection; * = mean of 400 determinations of 20 HM formulations.

**Table 4:** Concentration (mean ± SD) and prevalence (%P) of different aflatoxin types in samples of herbal medicine formulations collected from four districts of Punjab, Pakistan.

Punjabi Districts	AFB1		AFB2		AFG1		AFG2		AFs	
	Mean (±SD)	%P	Mean (±SD)	%P	Mean (±SD)	%P	Mean (±SD)	%P	Mean (±SD)	%P
Multan	0.59 (±1.14)	45	0.20 (±0.47)	32	0.718 (±2.23)	29	0.40 (±0.91)	27	1.913 (±3.57)	50
Bahawalpur	0.50 (±1.0)	38	0.16 (±0.34)	30	0.78 (±2.28)	35	0.34 (±0.92)	22	1.79 (±3.44)	46
Rahim Yar Khan	0.59 (±0.83)	47	0.16 (±0.29)	31	0.86 (±2.28)	37	0.30 (±0.78)	25	1.91 (±3.05)	54
Dera Ghazi Khan	0.63 (±0.76)	55	0.27 (±0.43)	45	0.78 (±2.31)	42	0.52 (±1.43)	34	2.20 (±3.40)	60

Results are expressed as mean of X determinations ± standard deviation; no statistically significant difference was observed among means within the columns (*p* > 0.05). Abbreviations are as in Table 3 above.

**Table 5:** Numbers of samples (percentage) in which aflatoxin concentration exceeded the maximum tolerable levels (MTLs) of aflatoxin B_1_ (AFB_1_) and total aflatoxins (AFs) as per the European Union (EU) standards [56,57]. The table contains original results obtained in this study. The standards of 2 and 4 ng/g of the EU were only used for comparison purposes, and they can be found in different sources.

HM Formulation	EU Standards	
	AFB1 (2 ng/g) *	AFs (4 ng/g) *
Hazmina Plus	3 (15)	2 (10)
Safoof-e-Lal	0 (0)	18 (90)
Safoof Musaffi-e-Khoon	0 (0)	2 (10)
Safoof Mumanek Khas	1 (5)	1 (5)
Safoof Lecodine	0 (0)	0 (0)
Akhseer Pachish	0 (0)	0 (0)
Johar Hazim	0 (0)	0 (0)
Khameera Marvareed	0 (0)	0 (0)
Safoof-e-Thandak	1 (5)	1 (5)
Safoof-e-Supari Pak	1 (5)	1 (5)
Gond Katira	1 (5)	0 (0)
Safoof-e-Mughaliz	0 (0)	12 (60)
Safoof-e-Tabkhir	2 (10)	0 (0)
Allergex	1 (5)	11 (55)
Safoof-e-Jarian	1 (5)	2 (10)
Senna Maki	2 (10)	3 (15)
Kalvanji Hazim	3 (15)	0 (0)
Majoon Azaraqi	2 (10)	1 (5)
Alhazim	4 (20)	1 (5)
Habis	1 (5)	0 (0)
Total	23 (5.75)	41 (10.25)

* The EU MTL.

**Table 6:** Average daily dose (ng/kg bw/day) for AFB_1_ and total AFs via the consumption of Eastern medicines, i.e., medicinal herbal formulations, from Pakistan.

Medicine Type	AFB_1_				AFs			
	Children		Adults		Children		Adults	
	LB	UB	LB	UB	LB	UB	LB	UB
Hazmina plus	0.07	0.07	0.09	0.09	0.10	0.13	0.14	0.15
Safoof-e-lal	0.09	0.09	0.11	0.11	1.40	1.41	1.70	1.71
Safoof Musaffi-e-Khoon	0.02	0.02	0.02	0.02	0.05	0.06	0.05	0.05
Safoof Mumanek Khas	NS	NS	0.03	0.03	NS	NS	0.05	0.05
Safoof Lecodine	N\S	NS	0.02	0.02	NS	NS	0.03	0.04
Khameera Marvareed	0.01	0.03	0. 01	0.01	0.01	0.01	0.01	0.01
Safoof-e-thandak	0.07	0.07	0.06	0.06	0.15	0.15	0.14	0.14
Safoof-e-Supari pak	NS	NS	0.03	0.03	NS	NS	0.06	0.06
Gond Katira	0.06	0.06	0.08	0.03	0.10	0.11	0.13	0.14
Safoof-e-Mughaliz	NS	NS	0.003	0.01	NS	NS	0.14	0.15
Safoof-e-Tabkhir	0.03	0.03	0.03	0.03	0.06	0.06	0.05	0.05
Allergex	0.03	0.03	0.02	0.02	0.12	0.13	0.11	0.11
Safoof-e-Jarian	NS	NS	0.03	0.03	NS	NS	0.06	0.06
Senna Maki	0.04	0.05	0.04	0.04	0.13	0.14	0.12	0.13
kalvanji Hazim	0.06	0.06	0.05	0.01	0.07	0.08	0.63	0.07
Majoon Azaraqi	NS	NS	0.02	0.02	NS	NS	0.04	0.05
Alhazim	0.11	0.12	0.10	0.11	0.16	0.18	0.15	0.16
Habis	NS	NS	0.02	0.03	NS	NS	0.03	0.04
Total *	0.05	0.06	0.04	0.04	0.21	0.22	0.17	0.18

Akhseer Pachish and Johar Hazim Medicinal Herbal formulations were not considered for exposure assessment, as their aflatoxins concentrations were below the LOD; LB = lower bound scenario (censored numbers < LOD were given zero); UB = upper bound scenario (censored numbers <LOD were given LOD values and censored numbers <LOQ were given LOQ values); AFs = sum of AFB_1_, AFB_2_, AFG_1_, AFG_2_; NS = not studied for this age group (herbal medicine is not for consumption by that particular age group); * = average of all medicines.

**Table 7:** Evaluation of MOE and cancer risk (cancer case/10^5^/individuals) for total AFs via consumption of Eastern medicines.

Medicine Type	MOE				Cancer Risk per Year (per 75 Years)			
	Children		Adults		Children		Adults	
	LB	UB	LB	UB	LB	UB	LB	UB
Hazmina Plus	1628.37	1499.49	1195.14	1100.54	0.0019 (0.1425)	0.0025 (0.151)	0.0027 (0.2025)	0.0029 (0.2175)
Safoof-e-Lal	121.233	120.49	100.101	99.4949	0.0264 (1.951)	0.0268 (2.010)	0.0323 (2.4525)	0.0325 (2.455)
Safoof Musaffi-e-Khoon	3366.16	3024.12	3705.86	3329.31	0.00095 (0.0712)	0.0011 (0.0825)	0.00091 (0.0675)	0.00094 (0.0675)
Safoof Mumanek Khas	NS	NS	3334.9	3197.42	NS	NS	0.0009 (0.0675)	0.0011 (0.075)
Safoof Lecodine	NS	NS	4946.69	4317.46	NS	NS	0.00072 (0.0525)	0.00075 (0.0563)
Khameera Marvareed	26680.3	17409.1	29372.8	19166.0	0.00011 (0.0083)	0.00018 (0.0135)	0.0001 (0.0075)	0.0002 (0.0127)
Safoof-e-Thandak	1136.73	1118.68	1251.45	1231.58	0.00281 (0.213)	0.00285 (0.211)	0.0026 (0.195)	0.0029 (0.217)
Safoof-e-Supari pak	NS	NS	2995.59	2837.22	NS	NS	0.0011 (0.0825)	0.0014 (0.105)
Gond Katira	1734.89	1608.62	1273.31	1180.64	0.0018 (0.135)	0.0021 (0.157)	0.00254 (0.1875)	0.0027 (0.2025)
Safoof-e-Mughaliz	NS	NS	1180.43	1152.07	NS	NS	0.0027 (0.2025)	0.0028 (0.212)
Safoof-e-Tabkhir	3054.86	2927.9	3363.14	3223.38	0.0011 (0.0825)	0.0011 (0.0825)	0.0009 (0.0675)	0.0013 (0.075)
Allergex	1381.59	1351.77	1521.02	1488.19	0.0023 (0.1725)	0.0023 (0.1725)	0.0021 (0.1575)	0.0022 (0.165)
Safoof-e-Jarian	NS	NS	2823.26	2724.1	NS	NS	0.0011 (0.0825)	0.0012 (0.0885)
Senna Maki	1277.33	1211.76	1406.23	1334.05	0.0025 (0.1875)	0.0026 (0.195)	0.0023 (0.1725)	0.0024 (0.183)
Kalvanji Hazim	2462.57	2180.35	2711.09	2400.39	0.0013 (0.0975)	0.0015 (0.1125)	0.0012 (0.091)	0.0015 (0.075)
Majoon Azaraqi	NS	NS	4102.14	3619.13	NS	NS	0.00081 (0.061)	0.00089 (0.0667)
Alhazim	1035.65	958.386	1140.17	1055.1	0.0031 (0.2325)	0.0033 (0.2475)	0.0028 (0.213)	0.0032 (0.225)
Habis	NS	NS	5492.67	4496.38	NS	NS	0.0006 (0.045)	0.0007 (0.0525)
Total *	3989.06	3037.33	3995.33	3219.58	0.00402 (0.3017)	0.00421 (0.3155)	0.00324 (0.2431)	0.00341 (0.2557)

Eastern medicines having aflatoxins concentration < LOD (Akhseer Pachish and Johar Hazim) were not considered for exposure assessment; LB = lower bound scenario (censored numbers < LOD were given zero); UB = upper bound scenario (censored numbers < LOD were given LOD values and censored numbers < LOQ were given LOQ values); NS = not studied for this age group (herbal medicine is not for consumption by that particular age group); * = average of all medicines.

## Data Availability

Publicly shared in Figshare repository (DOI-10.6084/m9.figshare.16587551).
